# Relationship between childhood adversity and premenstrual syndrome: mediating role of alexithymia and psychological distress in medical students

**DOI:** 10.1186/s12905-026-04392-4

**Published:** 2026-05-21

**Authors:** Mahsa Abdollahpour Kahriz, Negin Bahri, Amir Sam Kianimoghadam, Abbas Masjedi Arani, Hedyeh Riazi

**Affiliations:** 1 Department of Clinical Psychology, School of Medicine, Shahid Beheshti University of Medical Science, Tehran, Iran; 2 Department of Midwifery and Reproductive Health, School of Nursing and Midwifery, Shahid Beheshti University of Medical Sciences, Tehran, Iran; 3 Department of Clinical Psychology, School of Medicine, Shahid Beheshti University of Medical Sciences, Tehran, Iran

**Keywords:** Adverse Childhood Experiences, Affective Symptoms, Premenstrual Syndrome, Psychological Distress, Students, Medical

## Abstract

**Objective:**

This study was conducted to identify the psychological factors that predict the severity of Premenstrual Syndrome (PMS) among young women in 2025.

**Methods:**

In this cross-sectional study, researchers selected 673 female medical students through convenience sampling. They collected data using the Childhood Trauma Questionnaire (CTQ), the Premenstrual Symptoms Screening Tool (PSST), the Toronto Alexithymia Scale (TAS-20), the Kessler Psychological Distress Scale (K10), and a demographic questionnaire. They analyzed the data using Structural Equation Modeling (SEM) in Amos and SPSS (Version 26).

**Results:**

The SEM model showed a good fit. PMS severity had significant positive relationships with psychological distress (B = 0.64, *p* < 0.001), childhood adversity (B = 0.262, *p* < 0.01), and alexithymia (B = 0.35, *p* < 0.001). A moderate positive correlation existed between alexithymia and PMS severity (*r* = 0.409, *p* = 0.01).

**Conclusion:**

Childhood adversity, alexithymia, and psychological distress significantly predict PMS severity. These findings highlight the importance of a biopsychosocial approach. We suggest that doctors, especially gynecologists, should consider these psychological factors when assessing and treating patients with PMS.

## Introduction

Premenstrual Syndrome (PMS) is a common condition that happens in cycles [1]. It includes a wide range of emotional, behavioral, and physical symptoms that occur in the luteal phase of the menstrual cycle and lessen with the start of menstruation [2]. Although up to 90% of women report some premenstrual symptoms, the rate of clinically significant PMS, those that meet specific diagnostic criteria, is much lower and varies by study. Estimates of clinically significant PMS range from 12% to about 48% [3]. For example, a meta-analysis reported an average prevalence of 47.8% across 17 countries [1], whereas a survey of Iranian university students found a prevalence of 70.8% [4–6]. This variation underscores the heterogeneity in PMS prevalence across different populations and assessment methods.

The etiology of PMS is complex, as it involves an interplay among physiological, psychological, and lifestyle factors. Beyond the symptoms themselves, PMS can significantly impair academic and occupational performance and social functioning [1]. Among psychological risk factors, childhood adversity is a crucial early-life stressor with enduring consequences. The World Health Organization defines childhood adversity as a serious public health concern consisting of physical, sexual, and emotional abuse, neglect, and exploitation, which can all impede an individual’s development and well-being [7]. Of paramount importance is an emerging body of evidence indicating that such childhood adverse experiences are strong predictors of more severe PMS symptoms in adulthood [8]. A proposed mechanism is that traumatic childhood experiences may result in dysfunction of adaptive developmental systems that regulate emotions and physiology. This dysregulation, in turn, heightens vulnerability to the cyclic hormonal and mood changes characteristic of PMS and thereby increases premenstrual symptom severity and functional impairment [9].

Another variable that childhood abuse can predict is alexithymia. People who have experienced adversity and childhood abuse, especially emotional abuse, tend to decrease emotional response, depression, inability to be independent, inability to trust others, emotional instability, or suicide and homicide [10]. According to multiple information theories (MIT), exposure to trauma in childhood can affect a child’s ability to express feelings, reflect, and communicate emotionally in the form of alexithymia [11]. Childhood adversity can be a factor affecting distress tolerance in adulthood. Distress tolerance is often defined as one’s ability to withstand negative and/or uncomfortable emotional states [12]. Childhood adversity increases the risk of developing a wide range of mental disorders in adolescence and adulthood [13]. On the other hand, alexithymia and psychological distress, which are formed as a result of childhood abuse, can determine the severity of PMS. Recent studies have shown that psychological factors such as stress [12], emotional dysregulation [14], personality traits [15], alexithymia [16], and psychological distress [17] predict the severity of PMS.

Although premenstrual syndrome (PMS) is very common, its causes are varied and not fully understood. Treatments often focus on relief rather than a cure. Compounding this issue is the ongoing denial by some clinicians that PMS is a genuine health concern, which highlights its underlying causes even more. Recent research suggests a link between negative childhood experiences, especially abuse, and more severe PMS in adulthood. This connection may involve psychological factors, such as alexithymia and psychological distress. However, the specific psychological processes that link early hardship to increased PMS symptoms are still largely unclear. It has not been tested whether alexithymia, or difficulty in identifying and expressing emotions, and psychological distress act as either sequential or parallel mediators in this connection.

This study introduces a new, mechanism-focused perspective on this link. By suggesting that alexithymia and psychological distress might mediate this relationship, we aim to go beyond simply showing a correlation. We want to shed light on the psychological pathway that could explain why childhood abuse leads to more severe PMS. This approach is fresh because it combines developmental trauma theory with psychosomatic medicine. It implies that problems in emotional processing, along with general distress, could be critical parts of the puzzle. Understanding this pathway is important for clinical practice. It could shift the focus from just managing symptoms to implementing targeted psychological treatments, such as emotion-regulation training and trauma-informed care, that might change the course of PMS for those affected. Therefore, this study aimed to: (1) investigate the connection between childhood abuse and PMS severity in a group of medical students—a high-stress population where these links might be stronger; and (2) formally test the new idea that alexithymia and psychological distress mediate this relationship sequentially.

## Method

### Participants

The participants in this study were medical students aged 18 to 35 who experienced PMS, recruited from universities across the country. The required sample size was calculated in advance using G*Power 3.1. For a linear multiple regression analysis with four predictor variables, a medium effect size (f² = 0.15) was chosen based on previous research. To reach a statistical power of 0.95 at a significance level of 0.05, the analysis showed that a minimum of 129 participants was needed. The current research is a cross-sectional descriptive study, and data collection was conducted online using Porsline software. The inclusion criteria for this research include students should be in the age range of 18 to 35 years, do not smoke cigarettes, have bleeding duration of 3 to 10 days in two months before the research, have regular menstruation with cycles of 21 to 35 day, do not have a known physical disease such as diabetes, seizure disorders, hyperthyroidism, etc. and do not use antidepressants, hormones and contraceptives. Exclusion criteria were as follows: (1) lack of collaboration with the behavioral disease counseling center (2), excessive fatigue that could impair the participant’s ability to provide reliable responses, and (3) failure to complete the questionnaires. All participants employed informed consent. The Ethics Committee of Shahid Beheshti University of Medical Science approved the implementation of this study.

### Measure

**Childhood Trauma Questionnaire – short form (CTQ):** The Childhood Trauma Questionnaire Short Form includes 28 items, 25 clinical and 3 validity items, measuring an individual’s experiences of child abuse and neglect, retrospectively. This questionnaire is a shortened version of the 70-item Childhood Trauma Questionnaire. Each item on the CTQ-SF was rated on a 5-point Likert Scale (1 = Never true, 2 = Rarely true, 3 = Sometimes true, 4 = Often true, 5 = Very often true). Therefore, the total score per subscale ranged from 5 to 25. The cumulative score on the whole CTQ-SF ranged from 25 to 125. The validity of this questionnaire in Bernstein’s research [18] using Cronbach’s alpha is 0.87, 0.86, 0.95, 0.89, 0.78 for emotional, physical, sexual abuse, emotional and physical neglect, respectively. Concurrent validity has been reported to range from 0.59 to 0.78 [18]. In the present study, the reliability of this questionnaire, assessed using Cronbach’s alpha, was 0.44.

**The premenstrual symptoms screening tool (PSST)** Consists of 19 items, divided into two domains: the 14 DSM-IV somatic and psychological markers of PMS/PMDD are included in the first domain, while five questions assessing the functional effects of premenstrual symptoms make up the second domain. Each item is rated according to a 4-point Likert scale (0 = absent; 1 = mild; 2 = moderate; 3 = severe). The following symptoms are included in the first domain: anger/irritability, anxiety/tension, tearfulness, depressed mood/hopelessness, decreased interest in work, reduced interest in home, reduced interest in social activities, difficulty concentrating, fatigue/lack of energy, overeating/food cravings, insomnia, hypersomnia, feeling overwhelmed, and physical symptoms (breast tenderness, headaches, joint/muscle pain, bloating, weight gain). The effects of these symptoms are rated in the second domain: work effectiveness or productivity, relationships with coworkers, relationships with family, social life activities, and home responsibilities [19]. According to the internal consistency test, this scale’s reliability proved sufficient (Cronbach’s alpha coefficient: 0.93). According to CVR and CVI, the content validity proved satisfactory (0.7 and 0.8, respectively) [20]. The internal consistency of this questionnaire in the current research, as measured by Cronbach’s alpha, was 0.83.

**Toronto Alexithymia Scale (TAS-20):** The TAS-20 is a self-report scale comprising 20 items. The TAS-20 is one of the most commonly used measures of Alexithymia. The TAS-20 has 3 subscales: difficulty describing feelings, difficulty identifying feelings, and externally-oriented thinking. Items are rated on a 5-point Likert scale, where 1 = strongly disagree and 5 = strongly agree. There are 5 negatively keyed items (4, 5, 10, 18, and 19). The total alexithymia score is the sum of responses to all 20 items, while the score for each subscale factor is the sum of the responses to that subscale. The TAS-20 uses cutoff scoring: equal to or less than 51 = non-alexithymia, equal to or greater than 61 = alexithymia. Scores of 52 to 60 = possible alexithymia. The reliability of this scale Demonstrates good internal consistency (Cronbach’s alpha = 0.81) and test-retest reliability (0.77, *p*<0.01). Research using the TAS-20 demonstrates adequate levels of convergent and concurrent validity. The 3-factor structure was found to be theoretically congruent with the alexithymia construct. In addition, it is stable and replicable across clinical and nonclinical populations [21]. Cronbach’s alpha coefficient for this questionnaire in the present study was 0.77, indicating its reliability.

**Kessler Psychological Distress Scale (K10):** The-Kessler Psychological Distress Scale-10 consists of 10 items to screen for mental illness in the general population, scored on a 5-point scale: None of the time (0); A little of the time (1); Some of the time (2); Most of the time (3), and All of the time (4). A typical item is, “During the last 30 days, about how often did you feel nervous?” Kessler, et al. It consists of 10 affirmative items such as ‘During the past 30 days, about how often did you feel nervous?’ and ‘During the past 30 days, about how often did you feel that everything was an effort?’. It uses a 5-point Likert scale to register responses, where 1 is “None of the time” and 5 is “All of the time”. A total score is calculated from all responses [22]. The internal consistency of this questionnaire in the current research, as measured by Cronbach’s alpha, was 0.92.

### Permissions for questionnaires

All instruments used in this study are freely available for academic research purposes and were used in accordance with their respective guidelines. The Childhood Trauma Questionnaire-Short Form (CTQ-SF) is an open-access instrument developed by Bernstein and colleagues [18] and is widely used in research without requiring individual permission, provided that proper citation is given to the original developers. The Premenstrual Symptoms Screening Tool (PSST) was designed by Steiner and colleagues [23] as a screening instrument for research and clinical purposes and is freely accessible for non-commercial research. The Toronto Alexithymia Scale (TAS-20) is a standardized instrument that is freely available for research use when appropriately cited [21]. The Kessler Psychological Distress Scale (K10) is in the public domain and requires no permission for research use [22]. All instruments were used with proper citation to their original validation papers as indicated in the reference list.

### Procedure

The present study was approved by the Ethics Committee of Shahid Beheshti University of Medical Sciences (Approval ID: IR.SBMU.MSP.REC.1403.436). This study follows a cross-sectional design, and the statistical method is Structural Equation Modeling (SEM). The questionnaires were uploaded to the Porsline website. Then, the questionnaire link was provided to students at medical sciences universities aged 18 to 35.

### Statistical analysis

Descriptive statistics and preliminary analyses were done using SPSS version 26. Pearson’s correlation analysis examined the relationships among the study variables: childhood adversity, PMS severity, alexithymia, and distress tolerance. A p-value of less than 0.05 was considered statistically significant for all tests. Some missing values, which accounted for less than 1% of all data points, were imputed using the series mean method at the subscale level. The main analysis tested the proposed mediation model using path analysis in AMOS.

## Results

The present research includes 673 of Iranian medical students. The average age and standard deviation of age are 22.81 and 3.90, respectively. 8.9% of the participants were married, and 91.1 were single (Table 1).

**Table 1:** The characteristics of the study samples

Variable	(*n* = 673) *N*(%)
Age (Mean ± SD)	22.81 ± 3.90
Height (Mean ± SD)	163.67 ± 5.58
Weight (Mean ± SD)	61.49 ± 11.59
Educational level	
BSc	372 (54.7%)
MSc	76 (11.1%)
MD	216 (32.1)
PhD	14 (2.1%)
Economic status	
Good	161 (23.6%)
Intermediate	486 (71.8%)
Poor	31 (4.6%)
Marital status	
Single	617 (91%)
Married	61 (9%)
Age of onset of menstruation (Mean ± SD)	12.73 ± 1.40
Interval between periods of menstruation	
≤ 21 days	N (4.9%)
21–35 days	N (86.3%)
≥ 35 days	N (8.8%)
Length of menstruation	
≤ 5 days	N (14.6%)
5–7 days	N (74.4%)
≥ 7 days	N (11%)

According to Table 2, the standard deviations and Pearson correlation coefficients among the study variables. All variables were significantly correlated. The strongest correlation was between alexithymia and PMS severity (*r* = 0.409, *P* = 0.01).

**Table 2:** Mean, Standard Deviation, and Interco relations for Study Variables

Variable	M	SD	Correlations			
			1	2	3	4
1. Childhood adversity	39.72	9.31	1			
2. Psychological distress	19.77	9.93	0.383^**^	1		
3. اAlexithymia	55.97	12.12	0.321^**^	0.521^**^	1	
4. Severity PMS	31.98	8.36	0.297^**^	0.684^**^	0.409^**^	1

**Correlation is significant at the 0.01 level (2-tailed)

The results of the path analysis (Table 3) revealed a significant direct effect of psychological distress on PMS severity **(**β = 0.64, *p* < 0.001, 95% CI ). Higher psychological distress was associated with increased PMS severity. Childhood adversity also had a significant total effect on PMS severity (β = 0.26, *p* < 0.001, 95% CI). In other words, higher childhood adversity was associated with increased PMS severity. Furthermore, alexithymia was confirmed as a significant predictor in the model (β = 0.35, *p* < 0.001, 95% CI), in other words, as the severity of alexithymia increases, the severity of PMS also increases (Table 3).

**Table 3:** Path coefficients between childhood maltreatment variables, alexithymia, psychological distress and PMS severity

Effect				*P*-value
	Direct	**Indirect**	**Total**	
childhood adversity	0.03	0.262*	0.262*	0.001
psychological distress	0.64*	-	0.64*	
Alexithymia	0.07*	0.28*	0.35*	

The conceptual model was tested using LISREL. After setting up the structural comparisons, the aforementioned model was analyzed using the maximum likelihood method. The overall fit of the model was examined using fit indices. After analysis, the overall fit of the conceptual model was examined. The fit indices for the conceptual model are presented in Table 4. All indices met the recommended cutoff criteria, indicating an acceptable fit between the model and the observed data.Therefore, the structural model examining the mediating roles of Alexithymia and psychological distress in the relationship between childhood adversity and the severity of PMS provides an acceptable fit to the observed data. The goodness-of-fit indices for the conceptual model are presented in Table 4. The obtained values (χ²(320) = 1318.13, *p* < 0.001; CMIN/DF = 2.471; CFI = 0.97; IFI = 0.98; AGFI = 0.973; RMSEA = 0.058) meet or exceed established cutoff criteria for model acceptance. Specifically, the CFI and IFI values above 0.95, combined with an RMSEA below 0.06 and a CMIN/DF ratio below 3, indicate that the hypothesized model provides an excellent and plausible representation of the observed relationships among the variables in the studied population.

**Table 4:** Goodness Fit model indices of fitness indexes for the conceptual model

Indices	χ^2^	df	*P* value	CMIN/DF	RMSEA	AGFI	PNFI	IFI	CFI
Model									
First-order	1318.13	320	0.001	2.471	0.058	0.973	0.922	0.98	0.97
Cut off	-	-	> 0.05	< 3	< 0.08	> 0.5	> 0.5	> 0.9	> 0.9

χ^2^ Chi-squared, *df *Degrees of Freedom, *CMIN/DF* Minimum Discrepancy Function by Degrees of Freedom Divided, *RMSEA* Root Mean Square Error of Approximation, *AGFI*  Adjusted Goodness of Fit Index, *PNFI* Parsimonious Normal fit Index, *IFI* incremental fit Index, *CFI* Comparative Fit Index

**Fig. 1:**
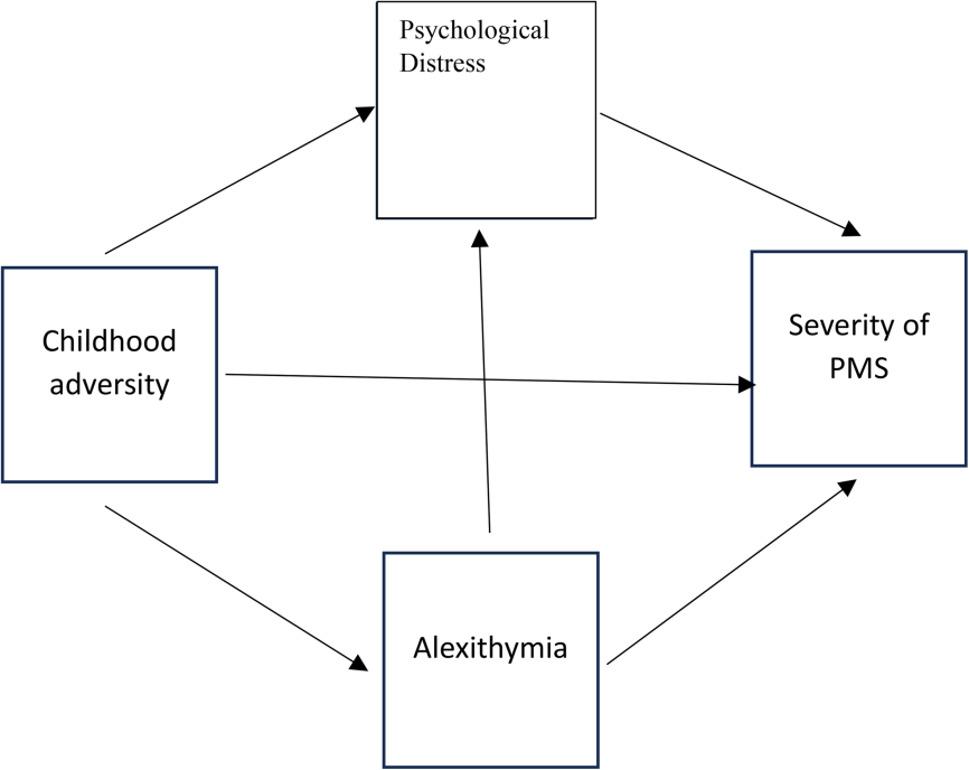
A path model for the relationships between the variables of childhood adversity, severity of PMS, Alexithymia and psychological distress

**Fig. 2:**
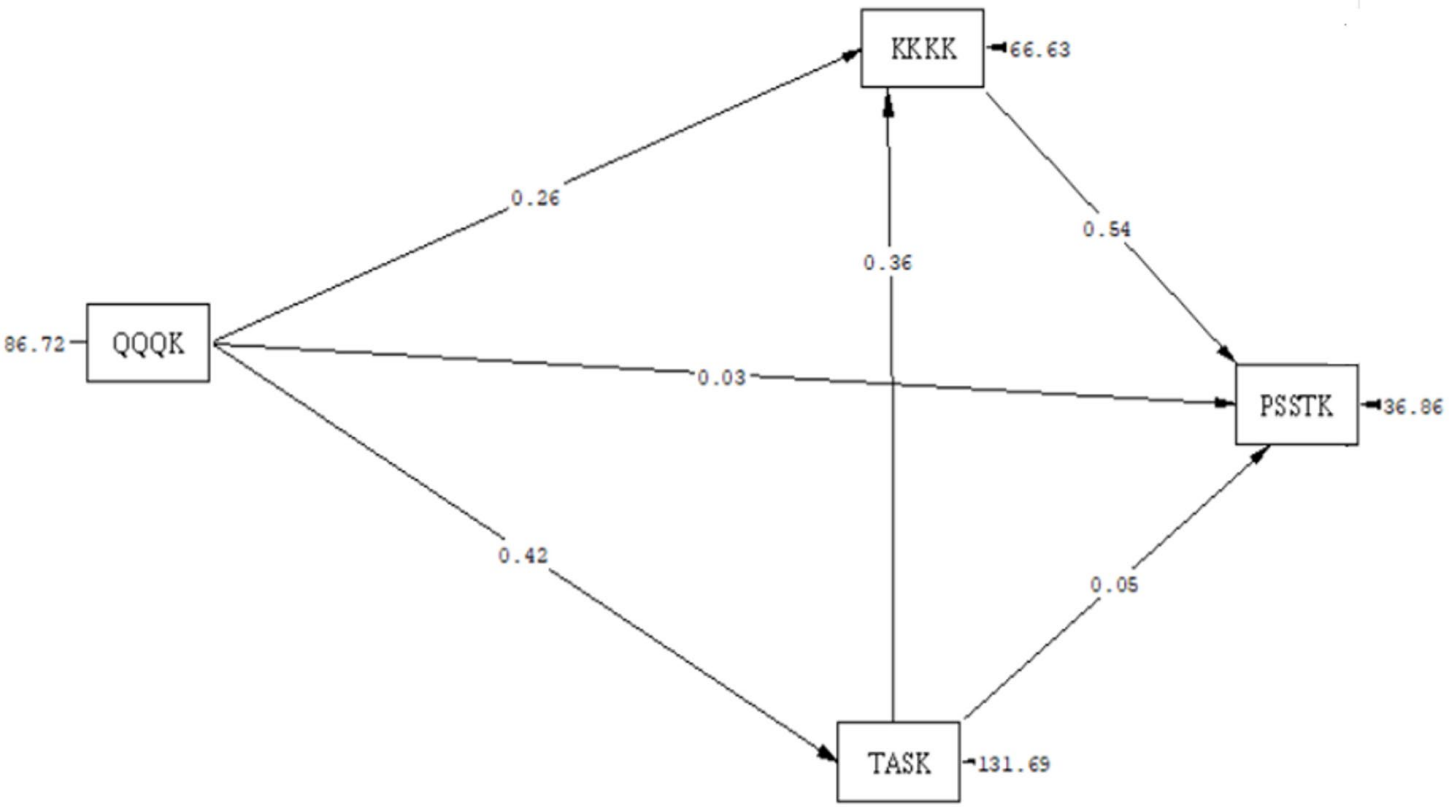
The initial structural model with the standardized coefficients

The results presented in Figs. 1 and 2 show that the path from childhood adversity to PMS severity is significant (t = 0.03, *p* < 0.01). The path from childhood adversity to psychological distress is significant (t = 0.26, *p* < 0.01). The path from childhood adversity to Alexithymia is significant (t = 0.42, *p* < 0.01). The path from Alexithymia to psychological distress is significant (t = 0.36, *p* < 0.01). The path from psychological distress to severity of PMS is significant (t = 0.54, *p* < 0.01). The path from Alexithymia to PMS severity is significant (t = 0.05, *p* < 0.01). The model explained 69% of the variance in PMS severity (R² = 0.69), indicating a substantial proportion of variance accounted for by the predictors. Childhood adversity showed both direct and indirect effects on PMS severity through psychological distress and alexithymia.

## Discussion

This study aimed to explore the link between childhood hardship and the severity of premenstrual syndrome (PMS). It also looked at how alexithymia and psychological distress play a role among medical students aged 18 to 35. The proposed model accounted for 69% of the difference in PMS severity (R² = 0.69). The findings showed that childhood adversity significantly increases PMS severity, as well as levels of alexithymia and psychological distress. These results match earlier research in this area [24–26].

The results of this study show that childhood adversity is significantly linked to the severity of PMS. This finding matches what other research has shown, as negative childhood experiences are known risk factors for different mental and physical health issues later in life [27]. We can hypothesize that experiencing childhood trauma may hinder a woman’s ability to recognize, manage, and adjust to the physical and emotional changes before menstruation. This could increase premenstrual distress and functional challenges. Although the direct connection between childhood adversity and PMS has not been studied as often, more research supports this link [27, 28].

Our analysis revealed that childhood adversity was a robust predictor of both higher levels of alexithymia and greater psychological distress. This suggests that early trauma may simultaneously impair emotional processing and increase general psychological vulnerability, creating two parallel pathways that converge to exacerbate premenstrual symptoms. This finding supports a larger body of research that highlights the increased risk for various mental health issues after childhood abuse [29, 30]. Specifically, adults who experienced childhood maltreatment tend to have a higher rate of depression, bipolar disorder, generalized anxiety, substance use disorders, suicidality, and other psychiatric conditions [31]. Regarding alexithymia, some types of child maltreatment have been linked to its development in adulthood [32]. For example, studies have found significant connections between alexithymia and emotional neglect, while links to physical, emotional, or sexual abuse have been less clear [33, 34].

In addition, in this education, we examined the relationship between childhood adversity and psychological distress. The result of the study showed that there is a positive and significant relationship between these two variables which is consistent with other studies [25, 35]. have also been indicated to be related to lesser mental health in adults, including mood disorders, substance abuse anxiety, personality and eating disorders, post-traumatic stress, as well as suicidality [10, 36]. Research indicated that childhood traumas will cause long-term psychosocial problems. Exposure to certain childhood adversities is associated with emotional responsiveness to stress and deficits in socioemotional adjustment [37].

These results highlight a strong link between alexithymia and psychological distress. The fact that alexithymia is overrepresented in clinical groups points to its being a vulnerability factor for psychopathology. Indeed, alexithymia has been specifically identified as a vulnerability factor for the development of depressive disorders [32]. Overall as a general vulnerability factor for a wide range of both internalizing and externalizing problems in nonclinical samples [38]. Generally, this confirms the substantial body of evidence indicating that high alexithymia constitutes a common characteristic feature across many diagnoses, such as somatoform disorders [39], self-harm behaviors [40], PTSD, and conduct disorder [41].

Moreover, the greater the degree of PMS symptom severity, the stronger the association with psychological distress. The determinants of PMS symptom severity are complex and multifactorial, including personal, lifestyle, occupational, physiological, and social causes [42]. Of these, stress, especially from interpersonal relationships, is an established correlate of severe PMS symptoms [42]. Thus, studies have reported that a depressed mood, anxiety, and sleep disturbances significantly contribute to enhanced symptomatology of PMS [43].

Finally, alexithymia was directly related to the severity of PMS. High alexithymia individuals have difficulty recognizing their physical and emotional states, which can make them vulnerable to somatization and the exaggeration of physical discomfort [44, 45]. Simultaneously, their limited adaptive emotion regulation, which may impair coping with cyclic stressors such as premenstrual changes [46]. This is further exacerbated by the established interpersonal problems among alexithymia individuals, which can increase the severity of premenstrual distress within a relationship [16, 46].

These findings should be interpreted considering several limitations. First, the cross-sectional design precludes the establishment of causal relationships among the variables. While associations were identified, the direction of these relationships (e.g., whether X leads to Y or vice versa) cannot be confirmed, and the influence of unmeasured confounding variables remains possible. Second, the reliance on online self-reported data may introduce biases. These include selection bias, as individuals without reliable internet access or digital literacy were underrepresented, potentially limiting the generalizability of the findings. Furthermore, self-report measures are susceptible to social desirability bias and common method variance, which may inflate the observed correlations. Third, the study sample consisted exclusively of Iranian medical students. This specific cultural, educational, and likely socio-demographic context limits the extrapolation of the results to other populations, including non-medical students, other age groups, or individuals from different cultural backgrounds. The psychological measures used, though validated in some contexts, may not fully capture the constructs as experienced within this specific population without further cultural validation. Future research should employ longitudinal or experimental designs to explore causal pathways. Multi-center studies with more diverse samples, including various professional groups and cultures, are needed to test the generalizability of the model. Incorporating objective or multi-informant measures could also help mitigate the limitations associated with self-report data.

Despite these limitations, the present study provides valuable insights into the mediating mechanisms linking childhood adversity to PMS severity among Iranian medical students and offers a foundation for future research.

## Conclusion

Our research showed that the severity of PMS can be influenced by several psychological factors such as childhood adversity, alexithymia, and psychological distress. This study proposes that all physicians, especially gynecologists, should consider psychological factors in the assessment and treatment of patients with PMS.

## Data Availability

The datasets used or analyzed during the current study are available from the corresponding author on reasonable request.

## References

[1] Sanchez BN, Kraemer WJ, Maresh CM. Premenstrual syndrome and exercise: a narrative review. Women. 2023;3(2):348–64.

[2] Dózsa-Juhász O, Makai A, Prémusz V, Ács P, Hock M. Investigation of premenstrual syndrome in connection with physical activity, perceived stress level, and mental status—a cross-sectional study. Front Public Health. 2023;11:1223787. 37601197 10.3389/fpubh.2023.1223787 PMC10435248

[3] Nexha A, Caropreso L, de Azevedo Cardoso T, Suh JS, Tonon AC, Frey BN. Biological rhythms in premenstrual syndrome and premenstrual dysphoric disorder: a systematic review. BMC Womens Health. 2024;24(1):551. 39375682 10.1186/s12905-024-03395-3 PMC11457342

[4] Direkvand-Moghadam A, Sayehmiri K, Delpisheh A, Kaikhavandi S. Epidemiology of premenstrual syndrome (PMS)-a systematic review and meta-analysis study. J Clin Diagn research: JCDR. 2014;8(2):106. 24701496 10.7860/JCDR/2014/8024.4021 PMC3972521

[5] Ranjbaran M, Samani RO, Almasi-Hashiani A, Matourypour P, Moini A. Prevalence of premenstrual syndrome in iran: a systematic review and meta-analysis. Int J reproductive Biomed. 2017;15(11):679. PMC5780553 29404529

[6] Reilly TJ, Patel S, Unachukwu IC, Knox C-L, Wilson CA, Craig MC, et al. The prevalence of premenstrual dysphoric disorder: systematic review and meta-analysis. J Affect Disord. 2024;349:534–40. 38199397 10.1016/j.jad.2024.01.066

[7] Zarchev M, Grootendorst-van Mil NH, Bouter DC, Hoogendijk WJ, Mulder CL, Kamperman AM. Childhood adversity and psychopathology: the dimensions of timing, type and chronicity in a population-based sample of high-risk adolescents. Child Adolesc Psychiatry Mental Health. 2024;18(1):37. 10.1186/s13034-024-00727-x PMC10949567 38500125

[8] Yesildere Saglam H, Gürsoy E, Karakuş A. Impact of childhood trauma history on premenstrual syndrome in women of reproductive age: a cross-sectional study. J Eval Clin Pract. 2025;31(1):e14172. 39396250 10.1111/jep.14172 PMC11713844

[9] Dawson DN, Eisenlohr-Moul TA, Paulson JL, Peters JR, Rubinow DR, Girdler SS. Emotion‐related impulsivity and rumination predict the perimenstrual severity and trajectory of symptoms in women with a menstrually related mood disorder. J Clin Psychol. 2018;74(4):579–93. 28898408 10.1002/jclp.22522 PMC5847394

[10] Švecová J, Furstova J, Kaščáková N, Hašto J, Tavel P. The effect of childhood trauma and resilience on psychopathology in adulthood: Does bullying moderate the associations? BMC Psychol. 2023;11(1):230. 37568213 10.1186/s40359-023-01270-8 PMC10422767

[11] Chung MC, Chen ZS. Gender differences in child abuse, emotional processing difficulties, alexithymia, psychological symptoms and behavioural problems among Chinese adolescents. Psychiatr Q. 2020;91:321–32. 31900820 10.1007/s11126-019-09700-w

[12] Agbaje OS, Nnaji CP, Nwagu EN, Iweama CN, Umoke PCI, Ozoemena LE, et al. Adverse childhood experiences and psychological distress among higher education students in Southeast Nigeria: an institutional-based cross-sectional study. Archives Public Health. 2021;79(1):62. 10.1186/s13690-021-00587-3 PMC8086118 33926542

[13] Levis SC, Baram TZ, Mahler SV. Neurodevelopmental origins of substance use disorders: Evidence from animal models of early-life adversity and addiction. Eur J Neurosci. 2022;55(9–10):2170–95. 33825217 10.1111/ejn.15223 PMC8494863

[14] Eggert L, Witthöft M, Hiller W, Kleinstäuber M. Emotion regulation in women with premenstrual syndrome (PMS): explicit and implicit assessments. Cogn Therapy Res. 2016;40:747–63.

[15] Erenoğlu R, Sözbir ŞY. Are premenstrual syndrome and dysmenorrhea related to the personality structure of women? A descriptive relation-seeker type study. Perspect Psychiatr Care. 2020;56(4):979–84. 32488914 10.1111/ppc.12551

[16] Sadeghi A, Gharibi S, Saadat S. Predicting the premenstrual syndrome based on alexithymia and self-efficacy in women with migraine: a cross-sectional study. J Iran Med Council. 2022.

[17] Fernández MdM, Regueira-Méndez C, Takkouche B. Psychological factors and premenstrual syndrome: a Spanish case-control study. PLoS ONE. 2019;14(3):1–13. 10.1371/journal.pone.0212557 PMC6402625 30840651

[18] Bernstein DP, Stein JA, Newcomb MD, Walker E, Pogge D, Ahluvalia T, et al. Development and validation of a brief screening version of the childhood trauma questionnaire. Child Abuse Negl. 2003;27(2):169–90. 12615092 10.1016/s0145-2134(02)00541-0

[19] Steiner M, Macdougall M, Brown E. The premenstrual symptoms screening tool (PSST) for clinicians. Archives women’s mental health. 2003;6:203–9. 10.1007/s00737-003-0018-4 12920618

[20] Hariri FZ, Moghaddam-Banaem L, Siah Bazi S, Saki Malehi A, Montazeri A. The Iranian version of the Premenstrual Symptoms Screening Tool (PSST): a validation study. Arch Women Ment Health. 2013;16:531–7. 10.1007/s00737-013-0375-6 23974654

[21] Bagby RM, Parker JD, Taylor GJ. The twenty-item Toronto Alexithymia Scale—I. Item selection and cross-validation of the factor structure. J Psychosom Res. 1994;38(1):23–32. 8126686 10.1016/0022-3999(94)90005-1

[22] Kessler RC, Andrews G, Colpe LJ, Hiripi E, Mroczek DK, Normand S-L, et al. Short screening scales to monitor population prevalences and trends in non-specific psychological distress. Psychol Med. 2002;32(6):959–76. 12214795 10.1017/s0033291702006074

[23] Steiner M, Macdougall M, Brown E. The premenstrual symptoms screening tool (PSST) for clinicians. Archives women’s mental health. 2003;6(3):203–9. 10.1007/s00737-003-0018-4 12920618

[24] Özşahin Z, Ünver H, Santur SG. Relationship between adverse childhood experiences and premenstrual syndrome. Med Records. 2022;4(1):27–34.

[25] Corcoran M, McNulty M. Examining the role of attachment in the relationship between childhood adversity, psychological distress and subjective well-being. Child Abuse Negl. 2018;76:297–309. 29175733 10.1016/j.chiabu.2017.11.012

[26] Cerqueira A, Almeida TC. Adverse childhood experiences: relationship with empathy and alexithymia. J Child Adolesc Trauma. 2023;16(3):559–68. 37593064 10.1007/s40653-023-00520-6 PMC10427576

[27] Ito K, Doi S, Isumi A, Fujiwara T. Association between childhood maltreatment history and premenstrual syndrome. Int J Environ Res Public Health. 2021;18(2):781. 33477613 10.3390/ijerph18020781 PMC7831299

[28] Azoulay M, Reuveni I, Dan R, Goelman G, Segman R, Kalla C, et al. Childhood trauma and premenstrual symptoms: the role of emotion regulation. Child Abuse Negl. 2020;108:104637. 32768748 10.1016/j.chiabu.2020.104637

[29] Mansueto G, Cavallo C, Palmieri S, Ruggiero GM, Sassaroli S, Caselli G. Adverse childhood experiences and repetitive negative thinking in adulthood: a systematic review. Clin Psychol Psychother. 2021;28(3):557–68. 33861493 10.1002/cpp.2590

[30] Messman-Moore TL, Bhuptani PH. A review of the long-term impact of child maltreatment on posttraumatic stress disorder and its comorbidities: an emotion dysregulation perspective. Clin Psychol Sci Pract. 2017;24(2):154.

[31] Scott JG, Malacova E, Mathews B, Haslam DM, Pacella R, Higgins DJ, et al. The association between child maltreatment and mental disorders in the Australian Child Maltreatment Study. Med J Aust. 2023;218:S26–33. 37004186 10.5694/mja2.51870 PMC10952950

[32] Zdankiewicz-Ścigała E, Ścigała DK. Attachment style, early childhood trauma, alexithymia, and dissociation among persons addicted to alcohol: structural equation model of dependencies. Front Psychol. 2020;10:2957. 32038366 10.3389/fpsyg.2019.02957 PMC6993624

[33] Ditzer J, Wong EY, Modi RN, Behnke M, Gross JJ, Talmon A. Child maltreatment and alexithymia: a meta-analytic review. Psychol Bull. 2023;149(5–6):311. 37261746 10.1037/bul0000391

[34] Chung MC, Chen ZS. Gender differences in child abuse, emotional processing difficulties, alexithymia, psychological symptoms and behavioural problems among Chinese adolescents. Psychiatr Q. 2020;91(2):321–32. 31900820 10.1007/s11126-019-09700-w

[35] Kaloeti DVS, Rahmandani A, Sakti H, Salma S, Suparno S, Hanafi S. Effect of childhood adversity experiences, psychological distress, and resilience on depressive symptoms among Indonesian university students. Int J Adolescence Youth. 2019;24(2):177–84.

[36] Miu AC, Szentágotai-Tătar A, Balazsi R, Nechita D, Bunea I, Pollak SD. Emotion regulation as mediator between childhood adversity and psychopathology: a meta-analysis. Clin Psychol Rev. 2022;93:102141. 35219929 10.1016/j.cpr.2022.102141 PMC8960368

[37] Sheikh MA. The potential protective effect of friendship on the association between childhood adversity and psychological distress in adulthood: a retrospective, preliminary, three-wave population-based study. J Affect Disord. 2018;226:21–7. 28942202 10.1016/j.jad.2017.09.015

[38] Bordalo F, Carvalho IP. The role of alexithymia as a risk factor for self-harm among adolescents in depression–A systematic review. J Affect Disord. 2022;297:130–44. 34695502 10.1016/j.jad.2021.10.029

[39] Koch AS, Kleiman A, Wegener I, Zur B, Imbierowicz K, Geiser F, et al. Factorial structure of the 20-item Toronto Alexithymia Scale in a large sample of somatoform patients. Psychiatry Res. 2015;225(3):355–63. 25613660 10.1016/j.psychres.2014.12.013

[40] Lee WK. Psychological characteristics of self-harming behavior in Korean adolescents. Asian J psychiatry. 2016;23:119–24. 10.1016/j.ajp.2016.07.013 27969068

[41] Deborde A-S, Maury SV, Aitel S. Régulation émotionnelle chez des adolescents présentant des troubles des conduites et chez des témoins. L’Encéphale. 2015;41(1):62–9. 24703786 10.1016/j.encep.2014.01.002

[42] Li L, Lv X, Li Y, Zhang X, Li M, Cao Y. Development and validation of risk prediction model for premenstrual syndrome in nurses: results from the nurses-based the TARGET cohort study. Front Public Health. 2023;11:1203280. 37854248 10.3389/fpubh.2023.1203280 PMC10579606

[43] Prabhavathi K, Hemamalini R, Poornima K, Saravanan A. Study of psychological predictors and sleep quality in different grades of premenstrual syndrome. Natl J Physiol Pharm Pharmacol. 2018;8(3):353.

[44] Habibi Asgarabad M, Salehi Yegaei P, Jafari F, Azami-Aghdash S, Lumley MA. The relationship of alexithymia to pain and other symptoms in fibromyalgia: a systematic review and meta‐analysis. Eur J Pain. 2023;27(3):321–37. 36471652 10.1002/ejp.2064

[45] Pei J-H, Wang X, Ma T, Du Y, Dou X. Alexithymia in a Chinese patient with chronic pain and associated factors: a cross-sectional study. Pain Manage Nurs. 2023;24(4):e1–6. 10.1016/j.pmn.2023.01.003 36774311

[46] Norouzi N, Manouchehri M. the Relationship between premenstrual syndrome symptoms and alexithymia with hypochondria: mediating role of marital satisfaction in women attending a women’s clinic. Psychol Nexus. 2024;1(1):48–67.

